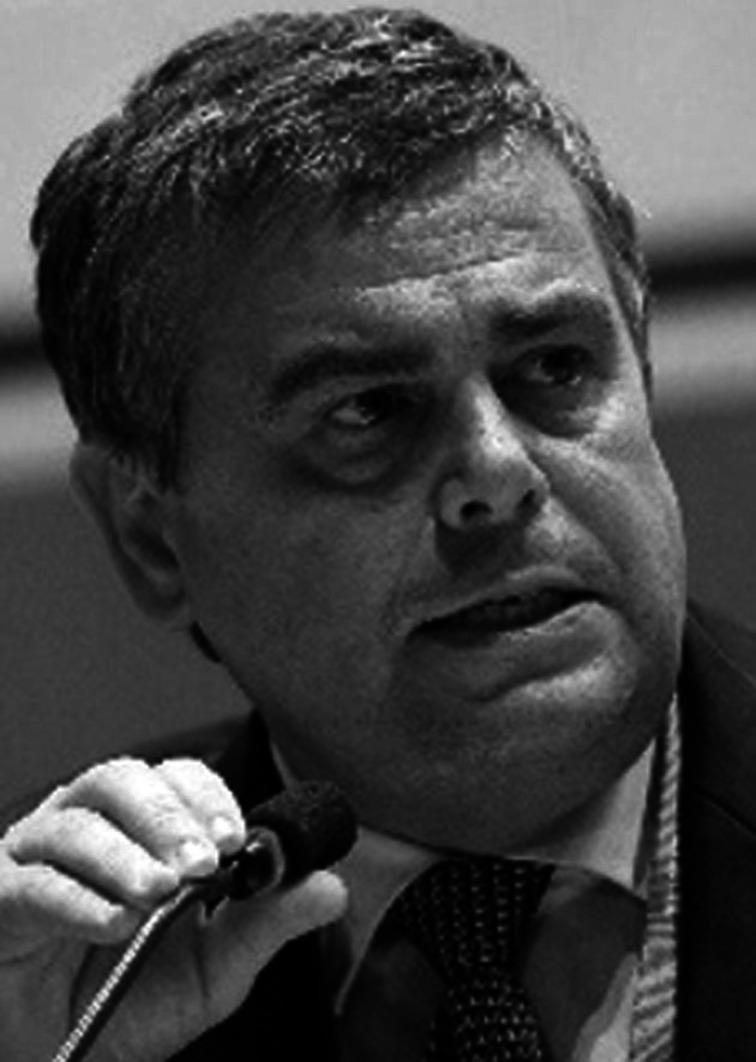# Across the Board: Gabriele Centi on Decoupling Electrocatalytic Reactions to Electrify Chemical Production

**DOI:** 10.1002/cssc.202200007

**Published:** 2022-01-27

**Authors:** Gabriele Centi

**Affiliations:** ^1^ Department ChiBioFarAm University of Messina Italy and European Research Institute of Catalysis (ERIC aisbl) Brussels, Belgium V.le F. Stagno D'Alcontres 31 98166 Messina Italy

**Keywords:** electrocatalysis, electrolysis, redox mediators, spatiotemporal decoupling, water splitting

## Abstract

In this series of articles, the board members of *ChemSusChem* discuss recent research articles that they consider of exceptional quality and importance for sustainability. This entry features Prof. G. Centi, who discusses the decoupling of the electrocatalytic reactions to realize spatiotemporal separation of the anodic and cathodic processes using redox mediators. This solution allows to potentially overcome the limitations due to intermittency of renewable energy production, besides a series of other advantages such as an improved energy efficiency.

## Graphical abstract

**Keep your distance**: Our board member Prof. Gabriele Centi discusses the decoupling of the electrocatalytic reactions to realize spatiotemporal separation of the anodic and cathodic processes using redox mediators. This solution allows to potentially overcome the limitations due to intermittency of renewable energy production.

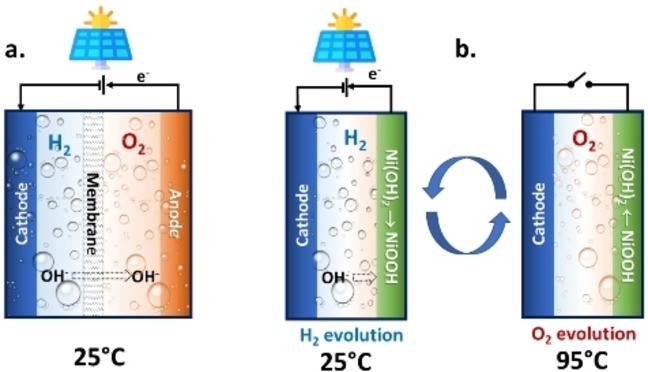

## Introduction

Moving towards a target of net zero emissions (by year 2050, as agreed politically for Europe) requires intensifying the development of technologies to substitute the use of fossil fuels, with electrification of the chemical production being one of the relevant targets. The largest part of the current chemical (and refinery) production is based on providing the energy needed for the chemical transformation in the form of heat, typically generated by converting fossil resources. Thus, a first mode to reduce the carbon footprint is to directly supply the heat via ohmic effect or by inductive, microwave, or radiofrequency heating. This approach could be called “indirect” because it mediates the effect via indirect heating. There are crucial issues of sector coupling between chemical production (requiring large and continuous supply of electrical energy in the case of electrification) and the intermittent renewable electrical energy production,^[1]^ as well as of storage and transport (to long distance) of the electrical energy, which limit the large‐scale introduction of this approach.

From the scientific perspective, most of the intrinsic limitations of the thermal approach still remain with this indirect electrification approach, such as the non‐selective supplying of the energy for the process and the possible presence of thermodynamic or heat transfer limitations. In addition, as the efficiency is often limited by heat exchange or recovery, large reactor volumes are requested, with energy integration becoming one of the relevant factors to push towards large‐size plants and intensive processes integration.^[2]^ This scale economy model is progressively losing the window of profitability due to the severe local environmental impact, large investments required (in an uncertain future), and limited flexibility in the production with respect to highly fluctuating demand. There are thus both social and industrial motivations to change the chemical production model from centralized to distributed,^[2]^ which has advantages in terms of integration with the local resources and territory, lower impact (better compatibility with self‐cleaning local capability of the environment), better flexibility to a very fast‐changing economic scenario, wider and different range of investors, and not least a better circularity and symbiosis with other production sectors. This transformation in the model of production, occurring in parallel to energy transition, requires changing the way through which the energy for the reaction is provided.[^3^, ^4^] In the direct mode of electrification, renewable electrical energy or sources are directly used to drive the chemical reaction.

From here, there is large and quickly increasing interest in the general area indicated as “reactive” catalysis (electro‐, photo‐, and plasma‐catalysis, to differentiate from “conventional” thermal catalysis).^[5]^ Electrocatalysis, among the methodologies of reactive catalysis, is that on which most of the industrial interest is currently focused. However, the actual performances are often still far from targets for industrial exploitation, even if continuous progresses must be mentioned. While research is focused mainly on electrodes, and in minor part on cell engineering, it should be remarked that the main limit of electrocatalytic (and in general of direct electrification) processes is instead the intermittency of the production of renewable energy. Continuous industrial operations are necessary to minimize fixed capital costs. If energy is taken from the grid, the current and next‐decade energy mix (up to 2035) will have a share of renewable energy below about 50 %. In these conditions, the carbon footprint of the electrocatalytic processes will be not acceptable, including that for electrolysis during hydrogen production. Thus, the challenge for electrocatalysis is to overcome this technological gap, rather than to improve only the electrocatalytic performance, the focus of most of the current worldwide research. High energy intensity (for water electrolysis ≈50 kW kg${}_{H{}_{2}}$ ^−1^) is another limit.

## Decoupling Electrocatalytic Reactions

Electrochemical processes are characterized from the presence of two separate reactions occurring at the cathode and anode sides, which should occur simultaneously. These two half‐cell reactions, however, can be decoupled by introducing redox mediators, as in flow batteries, either in solid state or dissolved in the electrolyte. This will realize a spatiotemporal separation of the anodic and cathodic reactions, providing an opening for many possible solutions. For example, designing a cell where the energy storage component operates in discontinuous modes (e. g., when renewable energy production is at a maximum), or by directly introducing a photo‐active component) and the cathodic reaction (e. g., CO_2_ electroreduction) in a continuous (24 h) mode, with the redox mediator accumulation acting as the buffer to implement this possibility. The advantage of this solution is the integration. For example, a photo‐electrocatalytic (PEC) device integrating a redox mediator can operate in a continuous way, solving the issues of intermittency of solar light. As an amortization of the fixed costs the crucial parameter in these devices, the possibility of continuous operations largely compensates the increased costs due to the introduction of the redox mediator. By introducing the latter, a spatial decoupling is also possible, thus allowing to search for new design integrations that optimize the performance.

**Figure 1:**
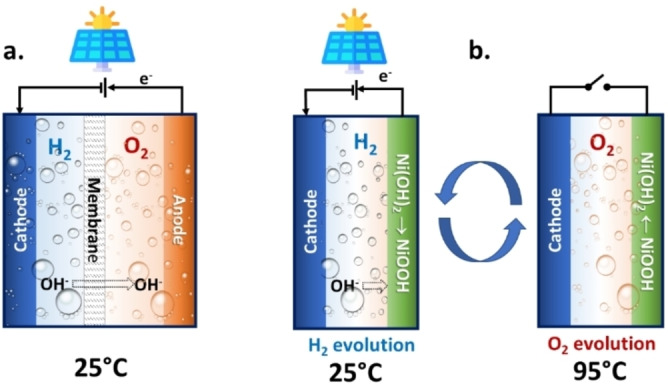
Schematic presentation of (a) conventional and (b) E‐TAC electrocatalytic water splitting device configurations. Redrawn from the original Figure in Ref. [20].

This decoupling of anodic and cathodic reaction is a current frontier of research, although with focus on water electrolysis.[^6^, ^7^, ^8^, ^9^, ^10^, ^11^, ^12^, ^13^] However, the spatiotemporal decoupling could be applied also to (i) PEC devices,[^6^, ^14^] leading to interesting solar‐to‐hydrogen conversion efficiency of 7.5 %, and (ii) other reactions than water electrolysis, such as direct conversion of N_2_ to ammonia.^[15]^ This is perhaps the most exciting direction to develop novel solutions for solar‐to‐energy vectors production in remote areas, to enable an effective renewable‐energy based society. The use of redox mediators allows not only spatiotemporal decoupling of anodic and cathodic reactions, but also brings about a series of additional potential benefits, such as better kinetics (e. g., with respect to oxygen evolution) and reduction of the overpotential, safety and operational flexibility, and not least a potential better control of competitive paths in reactions such as CO_2_ or N_2_ reduction. The redox mediators can be either in liquid phase (the electrolyte)^[16]^ or as solid‐state redox couple.^[17]^ A versatile solid‐state redox couple is Ni(OH)_2_/NiOOH. These materials, originally developed for rechargeable alkaline batteries, have been used for electrochemical–thermally‐activated chemical (E‐TAC) water splitting.[^18^, ^19^, ^20^] The interesting concept in E‐TAC is that the nickel hydroxide anode is charged (oxidized) to nickel oxyhydroxide (NiOOH), without concurrent oxygen evolution, at room temperature (25 °C). This stage is followed by a thermally activated chemical stage in which the cold alkaline electrolyte is replaced by a hot (95 °C) alkaline electrolyte to promote a spontaneous chemical reaction between the charged NiOOH anode and water that reduces the anode back to Ni(OH)_2_ while evolving oxygen (Figure 1).

Decoupling anodic and cathodic processes allows membrane‐free operation, reducing costs and eliminating crossover effects, which may significantly influence performance in the case of CO_2_ and N_2_ electrocatalytic reductions. In addition, a membrane‐less cell architecture allows easier high‐pressure operations. The E‐TAC approach presents another key advantage of high voltage and energy efficiency compared to both conventional electrolysis and to other decoupling strategies. Even if the standard potential of oxidation of Ni(OH)_2_ (1.42 V_RHE_, where RHE is the reference voltage of the reversible hydrogen electrode) is higher than that of the oxygen evolution reaction (OER, 1.23 V_RHE_), the one‐electron anode charging reaction shown in Equation (1) has no thermodynamic overpotential, in contrast to four‐electron OER requiring typical overpotential values in the 350–500 mV range (at a current density of 10 mA cm^−2^):

(1) $$\mathrm{Ni}\left(\mathrm{OH}\right){}_{2}+\mathrm{OH}{}^{-}\to \mathrm{NiOOH}+H{}_{2}O+e{}^{-}$$

In addition, operating the stage of oxygen evolution at higher temperature (Figure 1), there is a partial thermal contribution to the energy necessary for the overall process. The result is that the overall energy intensity of the process could be potentially reduced to 40 kW kg${}_{H{}_{2}}$ ^−1^ compared to about 50–55 kW kg${}_{H{}_{2}}$ ^−1^ in typical electrolyzers.^[18]^ A 95 % system efficiency is possible compared to about 70 % for alkaline and polymer electrolyte membrane electrolyzers.

However, this disruptive approach poses also significant challenges of materials and requirements for the electrodes. Doping with cobalt^[18]^ or traces of iridium^[21]^ shifts the oxidation cathodic potential by several tens of mV. The use of electrospun core–shell nickel/nickel hydroxide anodes^[19]^ and carbon‐cloth‐supported nickel hydroxide^[20]^ in the form of a nanoflake microstructure allows to improve mass transport and overall performance, besides stability. A further effort is necessary, however, to improve the behavior because both current density (≈10 mA cm^−2^) and capacity (the anode can be charged for ≈200 s) are still low, compared to redox mediators in liquid phase and, in general, to the need of achieving a spatiotemporal decoupling as that indicated above. Cost and separation of redox mediators in liquid phase, on the other hand, are also issues to solve.

## Conclusion

Decoupling the electrocatalytic reactions allows thus novel possibilities and to potentially solve the issue of intermittency of renewable energy production, besides other advantages, such as a better energy efficiency and enhanced design possibilities. However, many scientific challenges in terms of materials and device have yet to be solved to use this concept as a generalized solution, although the Israelian company H2Pro (www.h2pro.co) claims a commercial, megawatt‐scale product in 2023 with a 500 kg per day E‐TAC electrolyzer, operating at 50 bar.

## Conflict of interest

The authors declare no conflict of interest.

## Biographical Information

*Gabriele Centi is full professor of Industrial Chemistry at the University of Messina, Italy, and President of the European Research Institute of Catalysis (ERIC). He was coordinator of the EU Network of Excellence IDECAT and is President of IACS (International Association of Catalysis Societies). He recently started and coordinated an ERC Synergy grant on plasma‐catalysis. He is also part of the board of SUNERGY, the European initiative on solar fuels. He received several awards, such as the Chinese Academy of Science President's International Fellowship Initiative, PIFI, as Distinguished Scientist, and the Humboldt Research Award, and is involved in various editorial activities. He chaired the editorial board of* ChemSusChem *up to 2019 and is co‐editor in chief of* Journal of Energy Chemistry *and of the book series* Studies in Surface Science and Catalysis*. He was chairperson of many international conferences, such as Europacat 2017 in Florence and the 16th International Zeolite Conference. He is author of nearly 500 scientific publications, 12 books, and editor of over 20 special issues of journals. Current h‐index is 91 with over 32.500 citations (Google Scholar, Dec. 2021)*.